# Going beyond the mean: economic benefits of myocardial infarction secondary prevention

**DOI:** 10.1186/s12913-020-05985-x

**Published:** 2020-12-04

**Authors:** Viktor von Wyl, Agne Ulyte, Wenjia Wei, Dragana Radovanovic, Oliver Grübner, Beat Brüngger, Caroline Bähler, Eva Blozik, Holger Dressel, Matthias Schwenkglenks

**Affiliations:** 1 Department of Epidemiology, Epidemiology, Biostatistics and Prevention Institute, University of Zurich, Hirschengraben 84, 8001 Zurich, Switzerland; 2 Institute for Implementation Science in Health Care, University of Zurich, Universitätsstrasse 84, 8006 Zurich, Switzerland; 3 Department of Geography, University of Zurich, Winterthurerstrasse 190, 8057 Zurich, Switzerland; 4 Department of Health Sciences, Helsana Group, Zürichstrasse 130, 8600 Dübendorf, Switzerland; 5 Institute of Primary Care, University of Zurich and University Hospital Zurich, Pestalozzistrasse 24, 8091 Zürich, Switzerland

**Keywords:** Health care costs, Compliance, Causality, Costs and cost analysis, I10

## Abstract

**Background:**

Using the example of secondary prophylaxis of myocardial infarction (MI), our aim was to establish a framework for assessing cost consequences of compliance with clinical guidelines; thereby taking cost trajectories and cost distributions into account.

**Methods:**

Swiss mandatory health insurance claims from 1840 persons with hospitalization for MI in 2014 were analysed. Included persons were predominantly male (74%), had a median age of 73 years, and 71% were pre-exposed to drugs for secondary prophylaxis, prior to index hospitalization. Guideline compliance was defined as being prescribed recommended 4-class drug prophylaxis including drugs from the following four classes: beta-blockers, statins, aspirin or P2Y_12_ inhibitors, and angiotension-converting enzyme inhibitors or angiotensin receptor blockers. Health care expenditures (HCE) accrued over 1 year after index hospitalization were compared by compliance status using two-part regression, trajectory analysis, and counterfactual decomposition analysis.

**Results:**

Only 32% of persons received recommended 4-class prophylaxis. Compliant persons had lower HCE (− 4865 Swiss Francs [95% confidence interval − 8027; − 1703]) and were more likely to belong to the most favorable HCE trajectory (with 6245 Swiss Francs average annual HCE and comprising 78% of all studied persons). Distributional analyses showed that compliance-associated HCE reductions were more pronounced among persons with HCE above the median.

**Conclusions:**

Compliance with recommended prophylaxis was robustly associated with lower HCE and more favorable cost trajectories, but mainly among persons with high health care expenditures. The analysis framework is easily transferrable to other diseases and provides more comprehensive information on HCE consequences of non-compliance than mean-based regressions alone.

**Supplementary Information:**

The online version contains supplementary material available at 10.1186/s12913-020-05985-x.

## Background

Unwarranted variation in health care provision, reflected by deviation from treatment recommendations, is an ubiquitous problem and is associated with inefficient resource allocation, suboptimal treatment outcomes, lower quality of care, and higher health care expenditures (HCE) [1, 2]. However, to identify which health care services are ineffective or appropriate for a specific patient is challenging.

A crucial step towards improving efficiency of care is to establish a link between deviations from recommended care and inferior health and financial outcomes. Real-world studies of care provision are usually retrospective, observational, and relying on secondary data sources (that is, data initially collected for other purposes), which brings about risks of biases such as residual confounding [3]. Among the potential biases described in the literature the “healthy adherer bias” is of particular concern [4]. This bias circumscribes the effect that healthier persons tend to adhere better to prescribed treatments, for example because they are generally more health-conscious. Therefore, compliance may appear to exert beneficial effects on specific health outcomes when such benefits are driven by unmeasured comparator group differences.

Compliance has different facets: It can involve 1) prescription compliance of physicians with recommended guidelines, or 2) drug refill, or 3) intake by patients. Moreover, health care needs, as well as treatment compliance are often incompletely captured by routine databases (e.g. health insurance claims) because persons not accessing care are not recorded and standardized diagnostic information is often missing. Neither are actual drug intake by patients commonly part of administrative or health claims databases. Additionally, there is currently no established methodological framework for investigations into HCE implications of (non-)compliance with recommended health care. The limited scientific literature on the topic is dominated by mean-based methods, that is, cost outcomes are aggregated to total cost averages and analyzed in regression frameworks. While certainly valid and appropriate under clearly defined circumstances (e.g. cost-effectiveness studies nested in randomized trials), such approaches tend to discard valuable information regarding cost distribution, timing of clinical events, or the existence of subgroups “falling outside the norm”. Specifically, treatment recommendation compliance may not translate into health and cost benefits over the full disease-severity spectrum, but be limited to specific subgroups such as healthier persons without co-morbidities.

Therefore, this study aimed to revisit the effect of prescription and prescription fill compliance (as covered by health insurance claims databases) on different monetary and health outcomes, using the well-described example of secondary prevention of myocardial infarction (MI). Pharmacological prevention after acute MI events is, in most circumstances, considered standard of care by major treatment recommendations [5–8]. Treatment recommendations state that prophylactic treatment should be initiated after hospital discharge, ideally including drugs from 4 classes. In particular, prophylactic treatment should contain dual antiplatelet therapy (DAPT) including aspirin and a P2Y_12_ inhibitor (prasugrel, ticagrelor, or clopidogrel), lipid-lowering drugs, particularly high-intensity statins (STAT), angiotension-converting enzyme (ACE) inhibitors or angiotensin receptor blockers (ARB), and beta-blockers (BB). However, real-world observations suggest that patients are frequently also prescribed treatment combinations with less than four drug classes (e.g. 3-class treatments based on STAT, ACE inhibitors/ARB, and BB) [5, 9].

The efficacy of these treatment combinations - in terms of prevention of further MIs, mortality, or MI-caused re-hospitalizations - has been demonstrated by randomized controlled trials. However, the real-life effectiveness, particularly in light of expectable imperfect compliance by physicians (in timely prescribing these drugs) and patients (by not taking all recommended drugs), as well as financial outcomes of taking secondary prevention, are much less explored [10]. Several studies have reported substantial net cost benefits (that is, overall reduced health care expenditures) among persons who complied with recommendations compared with non-compliers, despite compliers having accrued higher medication expenditures [3, 11–13]. Moreover, secondary prophylaxis has been associated with clinical benefits [9, 14, 15].

On the basis of health insurance claims data of persons who were hospitalized for an MI event, this study evaluated health and financial outcome differences between persons who were prescribed (and have filled prescriptions for) secondary prevention of MI as recommended by guidelines compared to others who were not. The database did not allow to determine, however, whether drugs were truly taken in by patients. Standard analyses were complemented by novel methods that cover additional outcome dimensions by looking at distributional cost differences and cost trajectory differences between compliance groups, thereby leading to further insights into the dynamics of health care provision.

## Methods

### Overall approach

**Fig. 1:**
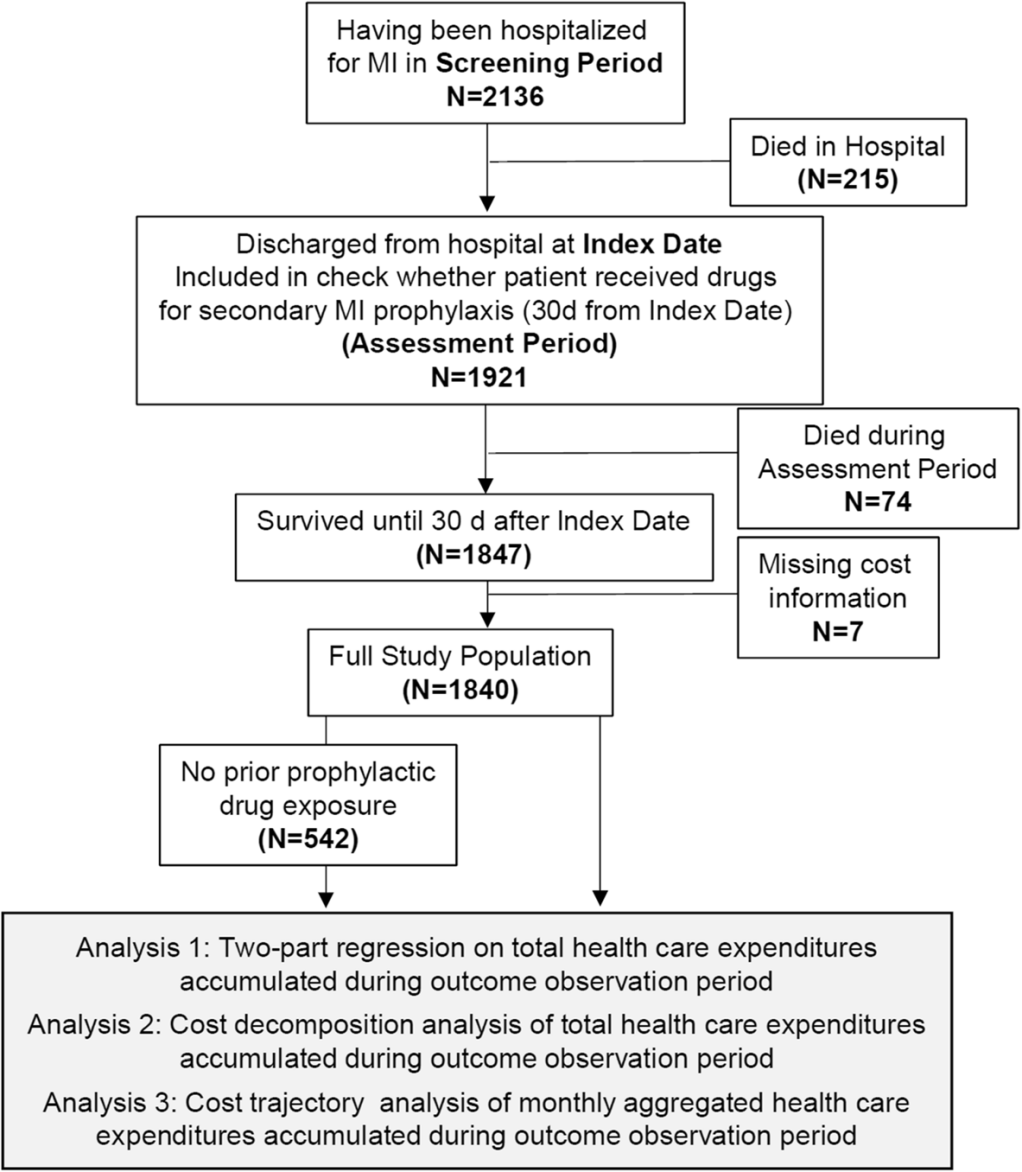
Study Flow Chart. Prior exposure was defined as having received P2Y12 inhibitors, ACE/ARB (angiotensin converting enzyme inhibitors/angiotensin-receptor blockers), Aspirin, or Beta blocker prior to Index date. Abbreviations: MI: Myocardial Infarction

This study evaluated the HCE implications of non-compliance to MI secondary prevention by applying recent methods from the causal inference framework to better control for healthy adherer bias (Fig. 1, analysis 1). HCE distributions (Fig. 1, analysis 2) and longitudinal perspectives (Fig. 1, analysis 3) were applied to provide a more comprehensive picture of (economic) consequences of compliance and to facilitate the investigation of subgroup-specific effects [16].

### Setting and location

All analyses were performed using health insurance claims data. Swiss mandatory health insurance characteristics are described elsewhere [17]. In brief, the insurance is comprehensive and reimburses costs using fixed fee (inpatient) and fee-for-service (outpatient) systems. There is a standard annual deductible of CHF 300 (that is, the insurance only covers HCE exceeding the deductible amount). Insurees can receive premium rebates for choosing higher deductibles (CHF 500 to 2500). Of note, the high deductibles and out-of-pocket payments of the Swiss system have been criticized for leading to foregone healthcare by some studies [18].

### Study population

The analysis is based on anonymized, administrative claims data from mandatory health insurance, provided by Helsana Insurance Group. This health insurer covers approximately 1.2 million people (15% of the full population), representative for the Swiss population. The database also included information on enrollees’ sociodemographic characteristics (including date of death), choice of insurance characteristics, as well as details on all reimbursed medical services (e.g. date of care provision, length of stay).

**Fig. 2:**
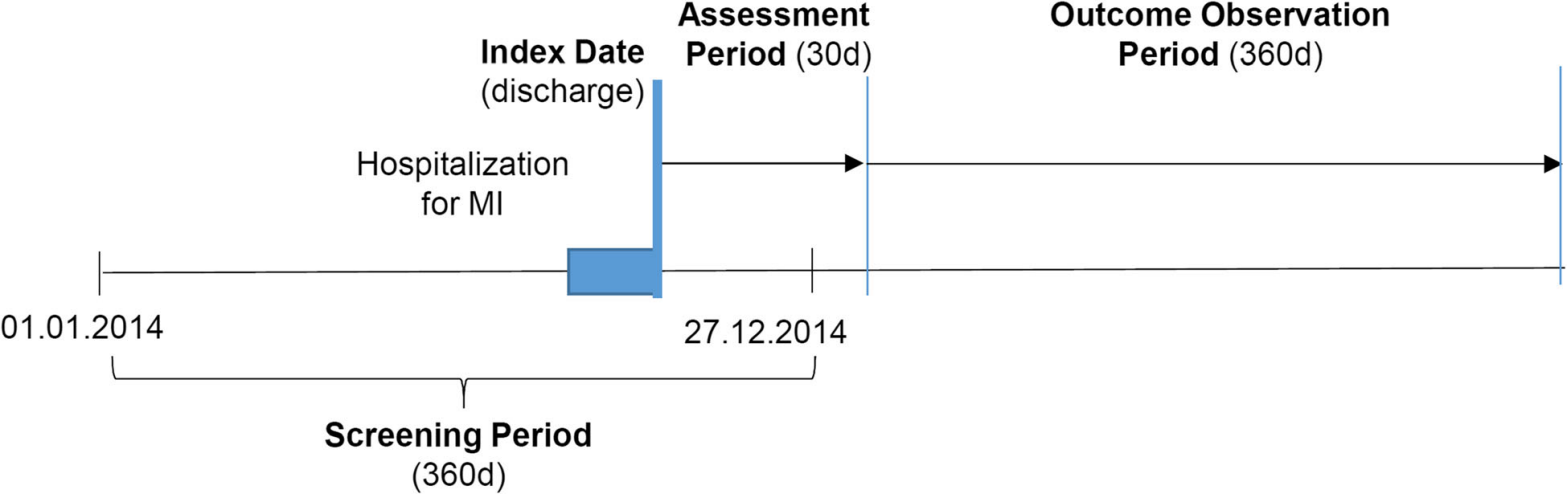
Study design and definitions of timelines. Abbreviations: MI: myocardial infarction; d: days

As illustrated in Fig. 2, this analysis included persons who were insured with Helsana during 2014 and 2015. Persons were selected if they were hospitalized with an MI (index hospitalization) until Dec. 27, 2014, as indicated by the International Disease Classification Version 10 (ICD-10) codes I21 (acute MI) and I22 (subsequent MI). We excluded patients with incomplete insurance coverage in 2014, asylum seekers, patients living outside Switzerland, Helsana employees, patients living in nursing homes and receiving lump-sum reimbursement (which could mask some services received), and those not surviving until the end of the assessment period (Fig. 2) [18].

### Study perspective and time horizon

This study analysed cost consequences from the view point of a Swiss health insurer. That is, only costs were considered that were reimbursable to insurees by mandatory health insurance. The cost contributions of Swiss cantons for inpatient stays (55% of inpatient costs) were not considered because they are handled directly between cantons and insurers and are therefore largely unaffected by insurance-scheme induced (dis-)incentives for compliance.

### Currency, price date, and conversion

All HCE are expressed in Swiss Francs (CHF), with 1 CHF being the equivalent of 0.92 Euros or 1 US $ (as of October 2019). Because all outcome analyses encompass a 360-day time-frame, no discount rates were applied.

### Ethics

Study data were anonymized before analysis. According to the national regulations, ethical approval was not required for this type of study.

### Outcome and explanatory variables

#### Economic outcome variables

This study analyzed HCE accrued during the outcome observation period (Fig. 2) from days 31 to 390 after discharge from the index hospitalization, considering all inpatient and outpatient services received during that period. HCE was categorized into outpatient treatment expenditures, inpatient care, drugs, and other costs (such as aids, home care). The primary outcome of the analyses was total HCE, but secondary analyses also looked into specific HCE categories (especially drug costs).

#### Health outcome variables

In addition, selected health outcomes were analyzed separately, namely deaths or hospitalizations occurring until 360 days during the outcome observation period (Fig. 2).

#### Main explanatory variable: compliance

The main explanatory variables were compliance to the recommended treatment, defined by reimbursement claims recorded within 30 days after the index date. The 30-day cut-off was chosen based on the reasoning that this time frame provided ample time for re-filling a prescription (as patients sometimes receive medications for a few days at hospital discharge).

Treatment recommendation compliance was defined as having filled one or more prescriptions for a 4-class combination therapy including STAT, BBs, ACE, and either aspirin or P2Y_12_ inhibitors. In sensitivity analyses, persons who received combination therapy containing at least 3 out of the 4 classes were also considered to be treatment recommendation compliant.

#### Other explanatory variables

All analyses considered socio-demographic, morbidity-, and insurance-related factors. Included variables comprised age, sex, living in a French- or Italian-speaking (as opposed to German-speaking) canton, urbanity of place of living (categorized as rural, sub-urban, or urban), having a high annual deductible of >CHF 500, having at least one supplementary health insurance, being in a managed care model, having pre-existing chronic morbidities requiring regular medication intake (as identified by pharmaceutical cost groups), having used anticoagulation drugs (heparin, vitamin K antagonists, athrombin [19]), having had inpatient stays of at least 3 days or high medication expenditures of at least CHF 5000 in the year prior to the baseline hospitalization. Pharmaceutical cost groups (PCG) are a widely employed means to reliably derive the presence of certain co-morbidities on the basis of prescriptions of disease-specific drugs (e.g. against HIV) [20].

Furthermore, any use of medications used for MI prevention before the index hospitalization (screening period, Fig. 2) was recorded. Study subjects were classified by pre-exposure based on prior use of the drugs, such as P2Y_12_ inhibitor, ACE, ARB, BB, but not high-intensity statins because these are frequently prescribed without a direct link to an elevated MI risk.

Because the majority of included patients (71%) were pre-exposed to MI prevention drugs (potentially indicating that the index hospitalization was not the first cardiovascular event), all analyses were performed on the full sample (*n* = 1840) and on a subset of patients without pre-exposure (*n* = 542).

### Statistical analysis

Three analyses were conducted to investigate different dimensions of cost differences (Fig. 1). Analysis 1 applied two-part models to annual HCE and medication-related HCE, to identify differences between the groups of compliers and non-compliers. Separate models were estimated for the two compliance definitions (4-class treatments and at least 3-class treatments in the main and sensitivity analysis, respectively). The choice of sensitivity analysis was motivated by a preliminary analysis of different exposure categories that indicated high numbers of 3-class combination prescriptions (Table 1).

**Table 1:** Baseline Characteristics

	All	not pre-exposed to prophylactic drugs	pre-exposed to prophylactic drugs
N	1840 (100%)	542 (100%)	1298 (100%)
**Demographics**			
Median age [interquartile range]	73.0 [61.5; 82.0]	61.0 [53.0; 73.0]	76.0 [67.0; 84.0]
Female sex	654 (35.5%)	157 (29.0%)	497 (38.3%)
Living in French/Italian speaking cantons (vs. Swiss German)	466 (25.3%)	136 (25.1%)	330 (25.4%)
Living in urban region (vs. rural/suburban)	1391 (75.6%)	413 (76.2%)	978 (75.3%)
**Insurance Characteristics**			
Annual deductible > 500 Swiss Francs	240 (13.0%)	147 (27.1%)	93 (7.2%)
Having supplementary insurance	1408 (76.5%)	398 (73.4%)	1010 (77.8%)
Having a managed care contract	741 (40.3%)	253 (46.7%)	488 (37.6%)
**Prior medication use for chronic co-morbidities**			
Cancer	33 (1.8%)	7 (1.3%)	26 (2.0%)
Cardiovascular diseases	1361 (74.0%)	63 (11.6%)	1298 (100.0%)
Type 1 or type 2 diabetes	382 (20.8%)	39 (7.2%)	343 (26.4%)
Hypertension	709 (38.5%)	48 (8.9%)	661 (50.9%)
Median number of chronic comorbidities [interquartile range]	3.0 [2.0; 3.0]	3.0 [2.0; 3.0]	3.0 [3.0; 3.0]
**High-intensity statin use prior index date**	694 (37.7%)	45 (8.3%)	649 (50.0%)
**Inpatient stays prior to index date**	428 (23.3%)	50 (9.2%)	378 (29.1%)
**High outpatient medication costs prior to index date**	154 (8.4%)	24 (4.4%)	130 (10.0%)
**Treatments received within 30 days after index date**			
Aspirin	1212 (65.9%)	455 (83.9%)	757 (58.3%)
P2Y_12_ inhibitors	1191 (64.7%)	406 (74.9%)	785 (60.5%)
ACE/ARB	1144 (62.2%)	366 (67.5%)	778 (59.9%)
Betablocker	1117 (60.7%)	358 (66.1%)	759 (58.5%)
High-intensity statins	990 (53.8%)	386 (71.2%)	604 (46.5%)
**Combination treatments received**			
Three drug classes^a^	486 (26.4%)	161 (29.7%)	325 (25.0%)
Four drug classes^a^	595 (32.3%)	236 (43.5%)	359 (27.7%)
**Clinical outcomes 390 days after index date (30 day assessment period and 360 day outcome observation period)**			
Having died after index date	175 (9.5%)	24 (4.4%)	151 (11.6%)
Having had inpatient hospital stays after index date	735 (39.9%)	146 (26.9%)	589 (45.4%)

^a^ Four class treatments include high intensity statins, beta-blockers, ACE/ARB and either Aspirin or P2Y12 inhibitors. Three class treatments only include three of the four drug classes

Two-part models consisted of a logit-part that models the probability of having non-zero HCE, based on covariates *x* for individual *i* [21, 22]. The second part included a generalized linear model that estimates the distribution of non-zero HCE.

$$ \left({\overline{HCE}}_i\right|\ {x}_i\left)=\Pr \left({HCE}_i>0\right|\ {x}_i\right)\times \Pr \left({\overline{HCE}}_i\right|{HCE}_i>0,{x}_i\Big) $$

After initial explorations, a gamma distribution and log-link was chosen for this analysis [21]. The two-part regression estimates were then back-transformed into Swiss Francs [23].

Initial analyses led us to speculate that some persons may not receive prophylactic medications as recommended due to reasons that also influence the outcome of interest (“healthy adherer bias”). Moreover, a non-negligible number of persons died during the observation period. To mitigate these potential problems, inverse probability weighted models were estimated [24]. Weights were derived from a multivariable logistic regression on having drug reimbursement claims that indicate compliance with 4-class treatments (or 3- and 4-class treatments in sensitivity analysis) within 30 days of the index date as well as a separate multivariable regression model for having died after the end of the assessment period and before or at end of the observation period. Death during the observation period may have affected our analysis of cumulative HCE in two ways: 1) deceased persons contributed less observation months to the analysis, and 2) end-of-life costs tend to be disproportionally high. Therefore, imbalances in death rates across treatment compliance arms may translate into biased estimates of HCE differences between compliance groups.

To mitigate these biases, person-specific weights were calculated as the inverse of the model-based predicted probabilities for receiving recommended drugs and for having died (whereby predictions from both regression models were multiplied to create a single weight). These combined weights were then applied to the two-part regression analysis.

We hypothesized that non-compliance will lead to statistically significantly higher HCE after adjustments for group differences, healthy adherer bias and censoring due to death during the observation period (Hypothesis 1).

Analysis step 2 addressed the issue of compliance effects possibly not being equal across the full HCE distribution (e.g. with an effect only visible in high-cost groups). Therefore, we applied a method for counterfactual decomposition to explore differences between the groups of compliant and non-compliant persons across the full HCE distribution [25]. This was achieved by estimating the quantile function of compliant and non-compliant persons across HCE distribution deciles (with 9 split points) and by adjusting for pre-specified covariates. HCE differences between compliant and non-compliant persons were then estimated per quantile, adjusted for population differences between the two groups [25]. Statistical significance was assessed by bootstrap-generated 95% confidence intervals.

We hypothesized that HCE differences between compliant and non-compliant persons are not homogenous across the full cost spectrum (Hypothesis 2).

In addition, analysis 3 compared longitudinal age- and sex-adjusted HCE trajectories between compliant and non-compliant groups, thereby only considering patients who survived the full observation period because the method does not allow for censoring [26]. Individual HCE trajectories of monthly aggregated, age- and sex-adjusted HCE were smoothed using fifth-degree polynomial linear regression and then k-means clustered, which produced a trajectory-based classification. The optimal number of trajectory groups was determined by an algorithm assessing explained variance and classification stability (supplementary Figure 1).

We hypothesized that the trajectory analysis would yield groups that are associated with unfavorable HCE profiles (e.g. high overall costs, substantial spikes) and that non-compliant groups would tend to congregate more in such high-cost groups (Hypothesis 3). Hypothesis 3 was specifically tested using multinomial logistic regressions, with trajectory groups treated as outcome variables, compliance status (yes/no) as variable of interest and confounder adjustments for variables described above.

## Results

### Study population

As illustrated by Fig. 1, 1,840 patients had a hospitalization due to an MI and were discharged alive. Of these, 1298 (70.5%) had pre-exposure to secondary prevention drugs, possibly suggesting that the index hospitalization did not represent their first cardiovascular event. Overall, 175 of 1840 (9.5%) persons died during the outcome observation period.

Table 1 illustrates further baseline characteristics, stratified by pre-exposure status. Of note, persons with pre-exposure were markedly older (median of 76 years compared to 61 years in the unexposed group), more frequently female (*n* = 497, 38% vs. *n* = 157, 29%), and had more pre-existing, medication-treated hypertension (*n* = 661, 51% vs. *n* = 48, 9%) and type 2 diabetes (*n* = 343, 26% vs. *n* = 39, 7%). Table 1 further shows data on compliance status, which – globally – varied from 54 to 66% for individual drugs. Uptake of 4- and 3-class combination prophylaxes was calculated at 32% (*n* = 595) and 27% (*n* = 486) of all patients, respectively.

Median [interquartile range] HCE for the observation period were CHF 6779 [2329; 18,390] for the full population, of which a median of CHF 1633 [778; 3028] for medication expenditures. In the subpopulation of previously unexposed persons, corresponding figures for HCE and medication costs were CHF 3455 [1638; 9631] and CHF 1058 [593; 1942], respectively.

### Description of health outcomes during outcome observation period

When grouping the full sample (*n* = 1840, including patients with pre-exposure) by compliance with 4-class combination therapy (3- or 4-class therapy in the sensitivity analysis, respectively), the number and percentage of persons dying during the outcome observation period was *n* = 154, 12% (*n* = 121, 16%) in the group of non-compliers (supplementary Table 1). By contrast, only *n* = 21, 3.5% (*n* = 54, 5%) of compliers died during the outcome observation period. Multivariable odds ratios from logistic regression also indicated a nearly 50% lower mortality among compliant persons (odds ratio in main analysis 0.52 [95% confidence interval 0.30; 0.91]; sensitivity analysis 0.64 [0.44; 0.92], supplementary Table 1).

Furthermore, the risk for re-hospitalization during the observation period was similar between compliers (main analysis: *n* = 217, 36.5%; sensitivity analysis: *n* = 397, 36.7%) and non-compliers (*n* = 518, 41.6% and *n* = 338, 44.5%, respectively, supplementary Table 1).

### Mean-based comparisons

Unadjusted total HCE differences for compliant and non-compliant persons are shown in Table 2, stratified by pre-exposure status to prophylactic drugs. Crude average HCE were CHF -5260 lower in persons receiving 4-class treatment. Moreover, the table presents results from standard two-part and inverse probability weighted models. In the main analysis, persons receiving 4-class combination treatment had overall HCE that were CHF -2144 lower compared to non-compliant persons in the unweighted, basic model. When applying inverse probability weights derived from multivariable logistic regression (supplementary Table 2), this difference increased to CHF -4865 and reached statistical significance, but was still smaller than the crude average difference. Estimated differences were somewhat lower when only analyzing persons without pre-exposure, with a crude average difference of CHF **-**2935, CHF -1708 in the unweighted and CHF -4048 in the weighted model, and did not reach statistical significance. HCE differences were nominally of similar (full population) or even larger (unexposed population) magnitude when considering the use of 3- or 4-class combination treatments as compliant (sensitivity analysis), but also did not reach statistical significance (Table 2).

**Table 2:** Mean-based comparisons of HCE between compliers to recommended secondary prevention (column headings) and non-compliers

	4-class combination (main analysis)	3- & 4-class combination (sensitivity analysis)
**Full population (*n*** **= 1840)**		
Compliers Median [IQR]	12,504 [11,061; 13,948] (*n* = 595)	13,942 [12,720; 15,164] (*n* = 1081)
Non-compliers Median [IQR]	17,764 [16,081; 19,447] (*n* = 1245)	19,084 [16,660; 21,508] (*n* = 759)
Crude difference, mean [95%CI]	**−5260 [− 7890; − 2630]**	**− 5142 [− 7640; − 2644]**
TwoPM, unweighted Predicted difference [95%CI]	−2144 [− 4956; 668]	− 2737 [− 6081; 606]
TwoPM, IPTW Predicted difference [95%CI]	**−4865 [−8027; −1703]**	− 3837 [− 8703; 1030]
**No prior exposure (*n*** **= 542)**		
Compliers Median [IQR]	8134 [6380; 9887] (*n* = 236)	8360 [7082; 9638] (*n* = 397)
Non-compliers Median [IQR]	11,069 [7319; 14,818] (*n* = 306)	13,708 [6037; 21,379] (*n* = 145)
Crude difference, mean [95%CI]	**−2935 [− 7466; − 1596]**	**− 5347 [−10,410; − 285]**
TwoPM, unweighted Predicted difference [95%CI]	−1708 [− 4688; 1273]	− 4389 [− 10,158; 1380]
TwoPM, IPTW Predicted difference [95%CI]	−4048 [− 8727; 632]	− 6974 [− 17,959; 4011]

HCE amounts represent Swiss francs (CHF).Two-part models were estimated for compliance status to a 4- or 3- and 4- class combination treatment (main variable of interest shown in the table) and adjusted for age, sex, having a high deductible, participating in a managed care model, having at least one supplementary insurance, living in a French-speaking or Italian-speaking canton, degree of urbanity of place of living, having had high medication expenditures of at least CHF 5′000 within 360 days before the index date, having had an inpatient hospital stay within 360 days before the index date (other than the index hospitalization for myocardial infarction), as well as the presence of pharmaceutical cost groups (co-morbidites) as confounders (coefficients not shown)

In addition, inverse probability for compliance to a specific drug combination (IPTW) was applied to further adjust for the “healthy adherer bias”. Inverse probability weights were estimated by means of a multivariable logistic regression with compliance with a specific drug (as indicated by the column heading) as outcome variable and the same variables as for the two-part model potential predictors

Abbreviations: *HCE* health care expenditures, *TwoPM* Two-part model, *IPTW* Inverse probability of treatment weights, *IQR* Interquartile Range, *95% CI* 95% Confidence Intervals. Estimates with CI not including 0 are in bold

Moreover, medication costs did not differ statistically significantly between compliance groups, but were nominally higher for compliers in the subgroup of persons without prior pre-exposure to prophylactic drugs (supplementary Table 3).

### Counterfactual distributional differences

**Fig. 3:**
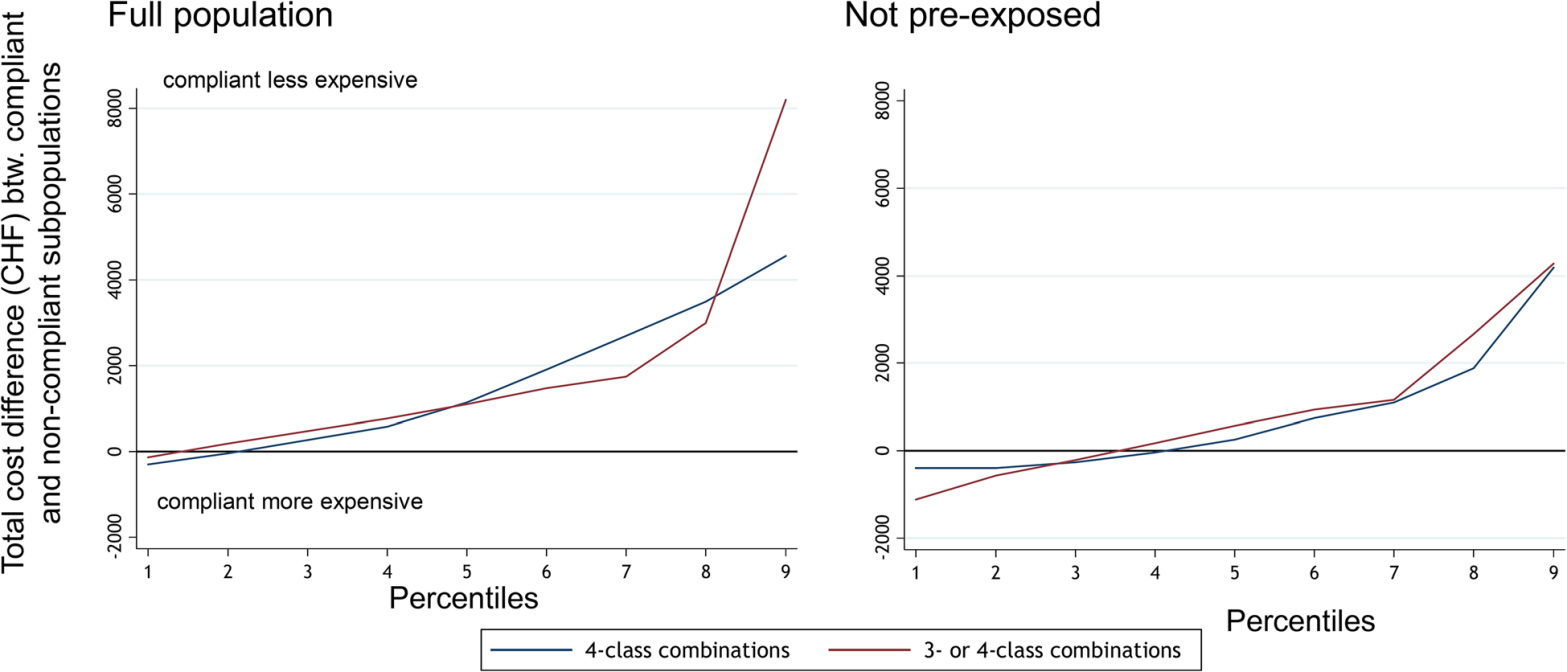
Health care expenditure differences between compliers and non-compliers across the full health care expenditure distribution. The decomposition analysis took the following potential confounders into account: age, sex, living in a French-speaking or Italian-speaking canton, degree of urbanity of place of living, having a high deductible, participating in a managed care model, having at least one supplementary insurance, having had an inpatient stay in the screening period (other than the index hospitalization for myocardial infarction), having had high medication expenditures of at least CHF 5000 in the screening period, number of pharmaceutical cost groups (which are drug-prescription based indicators for co-morbidities). Percentiles represent the 9 points in the HCE distribution that split the full sample into 10 equally large parts (deciles)

The results from the distributional analyses are presented in Fig. 3 and Table 3. Unadjusted distributions of HCE are displayed as split points for the HCE deciles in the second column of Table 3. For example, in the full population, the HCE deciles ranged from CHF 55 in the lowest to CHF 42,046 for the highest decile.

**Table 3:** Distributional cost composition analysis

Deciles	HCE	4-class combination (main analysis)	3- & 4-class combination (sensitivity analysis)
Full population (*n* = 1840)			
1	55	− 303 [− 659; 53]	−220 [− 732; 292]
2	1837	−90 [− 473; 293]	107 [− 397; 611]
3	2898	162 [− 345; 669]	379 [− 229; 988]
4	4386	510 [−164; 1184]	693 [− 106; 1493]
5	6779	**995 [33; 1958]**	982 [− 98; 2062]
6	10,062	**1730 [268; 3192]**	1304 [− 194; 2801]
7	15,135	**2504 [357; 4652]**	1475 [− 674; 3624]
8	23,369	**3516 [182; 6850]**	2291 [− 1341; 5923]
9	42,046	4984 [− 1360; 11,328]	6987 [− 823; 14,798]
Not pre-exposed (*n* = 542)			
1	629	− 340 [− 1074; 395]	− 1082 [− 5818; 3654]
2	1400	− 332 [− 836; 172]	− 479 [− 1779; 821]
3	1979	−315 [− 933; 303]	−72 [− 1250; 1105]
4	2544	− 132 [− 1007; 743]	328 [− 1258; 1914]
5	3455	202 [− 1124; 1529]	857 [− 1388; 3101]
6	4879	587 [− 1548; 2722]	1277 [− 2013; 4567]
7	7949	1200 [− 1968; 4367]	1716 [− 3359; 6792]
8	12,058	2087 [− 2436; 6610]	3881 [− 4434; 12,196]
9	21,928	4317 [− 2969; 11,603]	4233 [− 21,459; 29,924]

This table illustrates results from the counterfactual distribution analysis (total costs and costs attributed to compliance). Numbers in [square brackets] represent bootstrap-based 95% confidence intervals. HCE amounts represent Swiss francs (CHF). The decomposition analysis took the following potential confounders into account age, sex, living in a French-speaking or Italian-speaking canton, degree of urbanity of place of living, having a high deductible, participating in a managed care model, having at least one supplementary insurance, having had high medication expenditures of at least CHF 5′000 within 360 days before the index date, having had an inpatient hospital stay within 360 days before the index date, number of pharmaceutical cost groups (which are drug-prescription based indicators for co-morbidities). Deciles represent the 9 points in the HCE distribution that split the full sample into 10 equally large parts

Positive values indicate lower health care expenditures (HCE) in compliers, and vice versa

The counterfactual decomposition analysis was used to investigate HCE differences between compliance groups over the full HCE distribution (stratified by deciles), taking into account important confounders. In the main analysis, compliance-linked HCE differences were non-zero (indicating lower costs in compliant persons) and reached statistical significance from the fifth decile upward in the distribution (ranging from CHF 995 in the fifth to CHF 3516 in the ninth decile, left column in Table 3). The sensitivity analysis yielded qualitatively similar results that did not reach statistical significance. HCE differences were smaller among persons without pre-exposure but nominally also indicating smaller HCE in compliant persons in the second median half of the HCE distribution.

### Cost trajectories

**Fig. 4:**
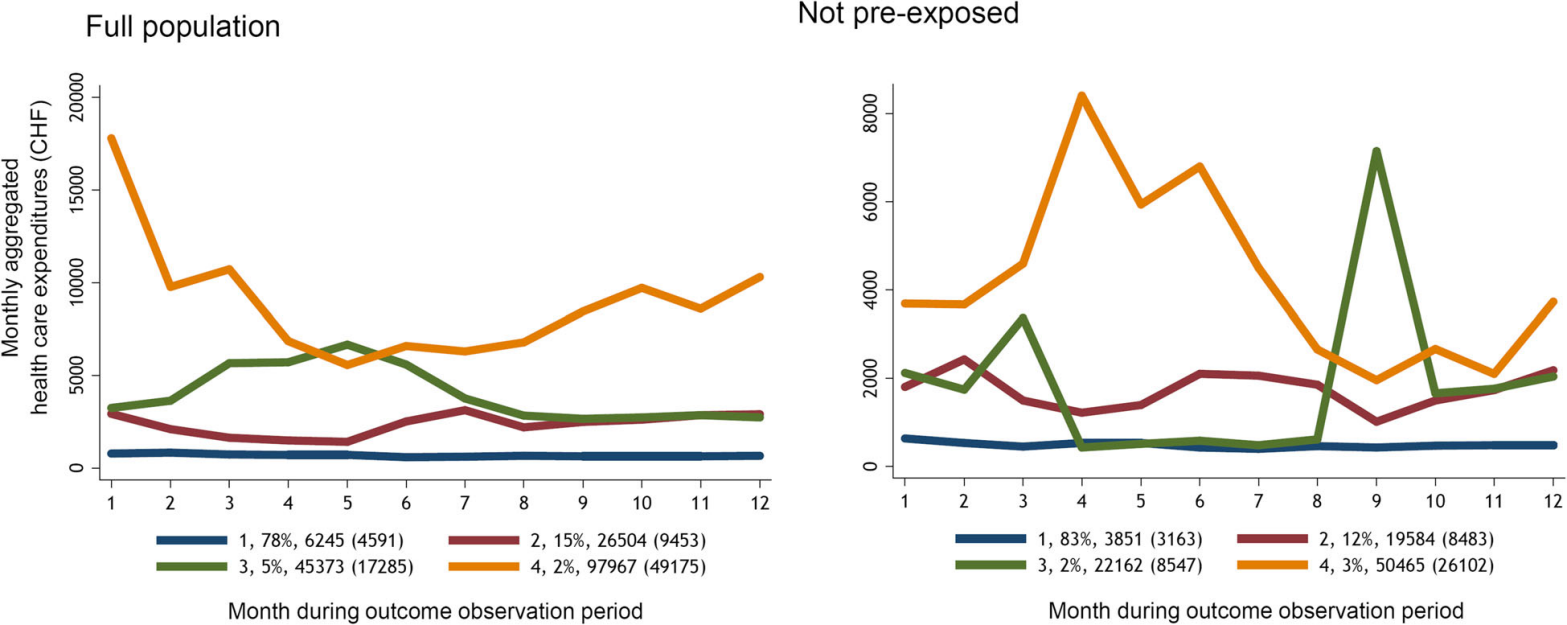
Cost trajectories. Numbers in figure legend indicate: trajectory group number, proportion of the analyzed sample, average total health care expenditures over full 12-month period (standard deviation)

The cost trajectory analysis was used to group patients into distinct classes, based on longitudinal HCE patterns over a one-year period after the end of the assessment phase (Fig. 4). The k-means based algorithm identified four robust groups, which were reflected both in the full and the previously unexposed groups. Parametrizations with fewer or more groups were inferior with respect to percent correctly classified and residual sums of squares (supplementary Figure 1).

In Fig. 4, the y-axis reflects average monthly HCE, the x-axis illustrates months since start of the outcome observation period. The analyses of the full and unexposed samples each suggested the presence of one large group (*n* = 1302, 78% and *n* = 429, 83%) with comparatively low average costs of CHF 6245 (full sample) and CHF 3851 (unexposed group) annually. The second largest groups comprised *n* = 245, 15% and *n* = 62, 12% of persons in the respective samples, but with substantially higher annual HCE of CHF 26,504 and CHF 19,584 and occasional peaks. The HCE spikes observed in group 2 (as well as in the high-cost groups 3 and 4) were largely driven by inpatient hospitalization costs (supplementary Figures 2 & 3), which constituted a high fraction of all HCE in all groups but the low-cost group 1.

On the basis of these findings, we evaluated our hypothesis that taking 4-class combination treatment (resp. 3- or 4-class therapies in the sensitivity analysis) as recommended may be linked to a higher probability of belonging to the more favorable low-cost group 1. The main analysis (Table 4) of the full population provided evidence for the hypothesis, as suggested by the overall *p*-value below the specified threshold of 0.05 and decreasing relative risk ratios below 1 (0.89, 0.71, and 0.20 for trajectory groups 2 to 4 when using the low cost group as reference, respectively). The latter indicates that, after controlling for important confounders, individuals taking 4-class combinations have a decreasing probability of belonging to one of the high-cost groups. Repeating the analysis for the sample of previously unexposed individuals, as well as when applying the sensitivity analysis definition of compliance, yielded inconclusive results however, mostly because numbers in high-cost trajectory groups were quite small.

**Table 4:** Comparison of cost trajectory groups

Prophylactic medication	Traj. Group	Full population N compliant (%)	Multivariable RRR [95%CI]	***p***-value	Not pre-exposed N compliant (%)	Multivariable RRR [95%CI]	***p***-value
4-class combination (main analysis)	1	478/1302 (36.7)	Ref.	**0.0191**	192/429 (44.8)	Ref.	0.5253
	2	71/245 (29.0)	0.89 [0.65; 1.22]		25/62 (40.3)	0.84 [0.47; 1.50]	
	3	22/87 (25.3)	0.71 [0.42; 1.21]		8/14 (57.1)	2.29 [0.67; 7.88]	
	4	3/31 (9.7)	0.20 [0.06; 0.71]		5/12 (41.7)	0.81 [0.24; 2.77]	
3- & 4-class combinations (sensitivity analysis)	1	353/1302 (27.1)	Ref.	0.5457	134/429 (31.2)	Ref.	0.4777
	2	65/245 (26.5)	1.07 [0.77; 1.48]		20/62 (32.3)	1.19 [0.65; 2.16]	
	3	28/87 (32.2)	1.44 [0.88; 2.34]		2/14 (14.3)	0.38 [0.07; 1.95]	
	4	7/31 (22.6)	0.96 [0.40; 2.34]		4/12 (33.3)	1.24 [0.34; 4.46]	

Distribution of compliers and non-compliers across the four trajectory groups (Fig. 4). This analysis investigates whether persons complying with a specific prevention drug combination have a higher probability of being included in the most favorable cost trajectory 1 (used as a reference). A multinomial logistic regression was used to test this hypothesis. The model was estimated for compliance status to a specific drug (main variable of interest shown in the table) and confounder adjustments as described in the methods section (coefficients not shown). *P*-values < 0.05 are highlighted in bold

Abbreviations: *RRR* multivariable Relative Risk Ratios, *95% CI* 95% Confidence Intervals, *Traj* Trajectory

Summary Table 5 illustrates the conclusions from the individual analyses. Recommended 4-class MI prevention was associated with robust HCE reductions in the full analysis sample, whilst compliance benefits among persons without prior exposure to MI prevention drugs were less clear, also due to a lack of statistical power.

**Table 5:**
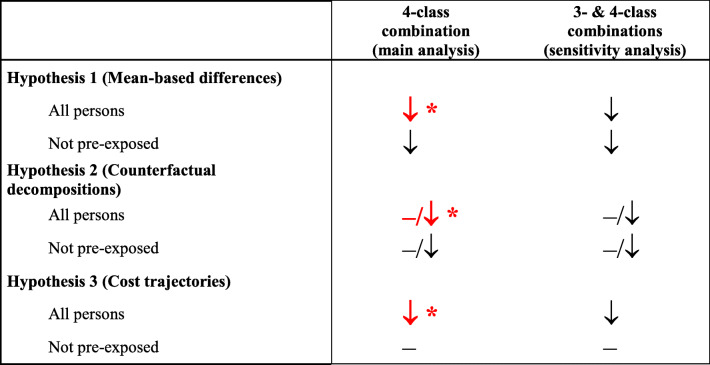
Summary of results

Summary of conclusions from Tables 2, 3 and 4. A minus (−) sign indicates no HCE reduction among compliant persons (meaning either no difference or even an increase.). Downward arrows indicate lower HCE in compliant persons, which have reached statistical significance < 0.05 if combined with a star (*) and in red bold. For the counterfactual decomposition analysis, the combination of symbols indicates that HCE differences become apparent only in the second median of the HCE distribution

## Discussion

Using reimbursement claims data from 1840 Swiss insurees, this analysis attempted to dissect economic consequences of non-compliance with pharmacological secondary prevention after a myocardial infarction. Using different methods to mitigate the healthy adherer bias and to explore effects of compliance across the full HCE distribution, we observed robust benefits of compliance for patients receiving 4-class combination treatment. As shown in Table 5, compliance with 4-class combination treatment was generally associated with statistically significant reductions and more favorable cost trajectory outcomes when analyzing all insurees. Moreover, compliance also went along with a markedly decreased risk for death during the observation period (that is, up to 390 days after index hospitalization discharge), but not for hospital readmission.

However, our sample included over 70% persons who likely had already experienced cardiovascular health problems prior to the index hospitalization and were pre-exposed to drugs for MI prevention. Therefore, we also performed sub-analyses on persons without pre-exposure, thereby assuming that the index hospitalization reflected the first myocardial infarction event. Although conclusions remained qualitatively the same (except in the trajectory analysis), the results no longer reached statistical significance, potentially due to the smaller sample size.

Overall, the findings fall well in line with previous research in the same field suggesting health benefits [9, 27] and lower health care expenditures for persons with a myocardial infarction who comply with secondary prevention recommendations [3, 11, 12, 28]. Although conducted in a Swiss setting, the findings are likely transferrable to target populations with similar demographics in other social health insurance schemes (e.g. Germany or the Netherlands).

From a methodological standpoint, the application of novel analytic approaches highlighted two important issues. First, the healthy adherer bias was indeed a confounding force and needs to be dealt with adequately in future studies. In particular, inverse probability weighted HCE difference estimates tended to be substantially larger than unweighted estimates; much so because a substantial number of persons died during the observation period, which was accounted for by our formulation of inverse probability weights. Of note, because our analysis omitted cantonal cost contributions to inpatient stay HCE, the estimates may even underestimate the actual HCE difference between compliant and non-compliant groups.

Moreover, the distributional analyses revealed that HCE reductions were larger between compliers and non-compliers as the overall level of HCE increased. Along the same lines, compliers had - after confounder adjustment - a greater likelihood for a more favorable HCE trajectory over 1 year after the assessment period than non-compliers (Table 4). These additional results complement the analysis of HCE means based on two-part regressions and suggest that cost benefits of compliance to 4-class combination therapy may in fact be driven by a prevention of costly complications (as indicated by a lower probability for compliers to belong to a high cost trajectory, as well as the increasing cost difference between compliers and non-compliers with rising overall HCE). Because inpatient hospitalizations are a major driver of HCE and hospitalizations were more frequently observed in the non-compliant group, one could speculate that better compliance with recommended secondary MI prophylaxis may translate into a lower risk for worsening of cardiovascular problems (possibly requiring hospital interventions). However, our database included limited information to substantiate this hypothesis, and these aspects clearly warrant further investigations as they may shed further light on the mechanisms of compliance-associated cost benefits.

The present analysis is – to our knowledge – one of the first to demonstrate a more differentiated cost effect of secondary MI prevention. Overall, the mix of methods we utilized has the potential to shed further light on the distribution and dynamics of HCE consequences of non-compliance. These methods have similar data requirements as standard multivariable analyses, can be implemented in standard software packages, and will easily translate to other disease domains as well. Furthermore, the findings have potential policy implications. Our results suggest that better compliance with secondary prevention treatments for myocardial infarction may lead to fewer health care expenditures. If further corroborated, this would imply that compliance with secondary MI prevention guidelines should be actively encouraged and monitored.

Some limitations need to be mentioned. Because of the observational nature of the analysis there remains a risk for residual confounding; all the more because the administrative database used only contains limited clinical information. Given this residual risk of bias, the observed cost differences between compliers and non-compliers should still not be considered causal, although they fall well within ranges observed by other studies. Further limitations are the restricted outcome observation period, the unavailability of information on drug intake by patients, as well as the static compliance definition based on a single time-point. Future analyses should, for example, include more detailed information on comorbidities, perform investigations into long-term outcomes and potentially develop more refined, dynamic measures of treatment compliance.

## Conclusions

By using novel analytical methods to examine distributions and trajectories of health care expenditures this study found that compliance with recommended secondary prevention consisting of 4-class combinations after myocardial infarction was associated with lower health care costs. The inclusion of methods for investigating the full dynamics and distribution of health care expenditures offers potential for more personalized cost-benefit analyses.

## Supplementary Information

**Additional file 1: Supplementary figure legend 1.** Indicators for trajectory model choice with respect to hypothesized number of distinct trajectory groups. The blue line indicates, for the validation data, the overlap between the training-based predictor and an independent k-means classification. Overall, classification overlap was in the order of 90% to 95% and peaked when four k-means groups were chosen. **Supplementary figure legend 2.** Cost decomposition by groups derived from the trajectory analysis, full sample. **Supplementary figure legend 3.** Cost decomposition by groups derived from the trajectory analysis, unexposed persons only. **Supplementary Table 1.** Comparison of clinical outcomes between compliers and non-compliers during observation period. **Supplementary Table 2.** Factors associated with compliance to 4-class secondary myocardial infarction prophylaxis (main analysis) or 3- or 4-class prophylaxis (sensitivity analysis). The results from the multivariable logistic regression model were used to calculate the inverse probability weights. Confidence intervals printed in bold face do not include 1, which indicates statistical significance at the 5% level. **Supplementary Table 3.** comparison of medication expenditures between compliers and non-compliers.

## Data Availability

The data underlying this study cannot be shared publicly because they are the property of Helsana (https://www.helsana.ch/en/helsana-group), and have restricted public access on grounds of patient privacy. The data are managed by Helsana and subsets of the database are available for researchers after request and under specific conditions. Data are available from Helsana (gesundheitskompetenz@helsana.ch) for researchers who meet the criteria for access to confidential data. Helsana will consider the possibilities of the research proposal and decide to grant access if the research questions can be answered with use of the Helsana data. When requests are granted, data are accessible only in a secure environment.
