# Supplementary material for: Going beyond the mean: economic benefits of myocardial infarction secondary prevention
Source: BMC Health Serv Res. 2020 Dec 4;20:1125. doi: 10.1186/s12913-020-05985-x (PMC7718707; doi:10.1186/s12913-020-05985-x)
Supplement: Supplementary file 1 — Additional file 1: Supplementary figure legend 1. Indicators for trajectory model choice with respect to hypothesized number of distinct trajectory groups. The blue line indicates, for the validation data, the overlap between the training-based predictor and an independent k-means classification. Overall, classification overlap was in the order of 90% to 95% and peaked when four k-means groups were chosen. Supplementary figure legend 2. Cost decomposition by groups derived from the trajectory analysis, full sample. Supplementary figure legend 3. Cost decomposition by groups derived from the trajectory analysis, unexposed persons only. Supplementary Table 1. Comparison of clinical outcomes between compliers and non-compliers during observation period. Supplementary Table 2. Factors associated with compliance to 4-class secondary myocardial infarction prophylaxis (main analysis) or 3- or 4-class prophylaxis (sensitivity analysis). The results from the multivariable logistic regression model were used to calculate the inverse probability weights. Confidence intervals printed in bold face do not include 1, which indicates statistical significance at the 5% level. Supplementary Table 3. comparison of medication expenditures between compliers and non-compliers. [file 12913_2020_5985_MOESM1_ESM.zip › Supplementary Materials_V2_cleanR2.docx]

***Supplementary Materials***

***Supplementary figure legend 1***

Indicators for trajectory model choice with respect to hypothesized number of distinct trajectory groups. The blue line indicates, for the validation data, the overlap between the training-based predictor and an independent k-means classification. Overall, classification overlap was in the order of 90% to 95% and peaked when four k-means groups were chosen.

The red line indicates how often a given cost trajectory, classified with the training-based predictor, would have been assigned to the same trajectory group on the basis of the least sums of squares criterion (calculated as the difference between the observed and the predicted trajectory on a given time point).

The green line indicates the R2 of a linear regression analysis including the k-groups and polynomials of the fifth degree (as used to estimate the person-based cost trajectories). Not surprisingly, R2 increases as the number of group k increases, but the green curve indicates differences in the additional amount of variance explained.

Given these three criteria, four groups were chosen as the best option for our analyses. Note that these criteria were analyzed independently for the sample of unexposed patients, which also yielded four groups as the best solution (not shown).

***Supplementary figure legend 2: Cost decomposition by groups derived from the trajectory analysis, full sample.***

The groups correspond to those illustrated in Figure 4 in the main manuscript (left panel)

***Supplementary figure legend 3: Cost decomposition by groups derived from the trajectory analysis, unexposed persons only.***

The groups correspond to those illustrated in Figure 3 in the main manuscript (right panel)

***Supplementary table 1: Comparison of clinical outcomes between compliers and non-compliers during observation period***

The multivariable logistic regression model includes compliance recommended 4-class combinations (c.f. column headings), as well as age, sex, high deductible, participation in managed care models, living in a French- or Italian- speaking canton, degree of urbanity of living place, having at least one supplementary insurance, having had high medication expenditures of CHF 5’000 in the screening period, having had inpatient hospital stays in the screening period, and the presence of pharmaceutical cost groups (co-morbidities) as confounders.

Abbreviation: mOR: multivariable Odds Ratio; 95% CI: 95% Confidence Intervals

|  | **4-class combination (main analysis)** | **3- & 4-class combination (sensitivity analysis)** |
| --- | --- | --- |
| **Death during outcome observation period** |  |  |
| **Full sample** |  |  |
| Non-complier, n (%) | 154/1245 (12.4) | 121/759 (15.9) |
| Complier, n (%) | 21/595 (3.5) | 54/1081 (5.0) |
| Multivariable logistic regression, mOR [95% CI] | **0.52 [0.30; 0.91]** | **0.64 [0.44; 0.92]** |
| **Subgroup without prior exposure** |  |  |
| Non-complier, n (%) | 18/306 (5.9) | 17/145 (11.7) |
| Complier, n (%) | 6/236 (2.5) | 7/397 (1.8) |
| Multivariable logistic regression, mOR [95% CI] | 0.66 [0.25; 1.77] | **0.27 [0.10; 0.69]** |
| **Hospitalization during outcome observation period** |  |  |
| **Full sample** |  |  |
| Non-complier, n (%) | 518/1245 (41.6) | 338/759 (44.5) |
| Complier, n (%) | 217/595 (36.5) | 397/1081 (36.7) |
| Multivariable logistic regression, mOR [95% CI] | 1.00 [0.80; 1.25] | 0.91 [0.74; 1.11] |
| **Subgroup without prior exposure** |  |  |
| Non-complier, n (%) | 86/306 (28.1) | 43/145 (29.7) |
| Complier, n (%) | 60/236 (25.4) | 103/397 (25.9) |
| Multivariable logistic regression, mOR [95% CI] | 0.97 [0.64; 1.46] | 0.96 [0.61; 1.51] |

***Supplementary Table 2***

Factors associated with compliance to 4-class secondary myocardial infarction prophylaxis (main analysis) or 3- or 4-class prophylaxis (sensitivity analysis). The results from the multivariable logistic regression model were used to calculate the inverse probability weights. Confidence intervals printed in bold face do not include 1, which indicates statistical significance at the 5% level.

|  | **4-class (main analysis)** | | | **3-4 class (sensitivity analysis)** | | |
| --- | --- | --- | --- | --- | --- | --- |
|  | **Non-compliant (n=1245)** | **Compliant (n=595)** | **Multivariable Odds Ratio [95% confidence interval]** | **Non-compliant (n=759)** | **Compliant (n=1081)** | **Multivariable Odds Ratio [95% confidence interval]** |
| Age | 75 [64;84] | 67 [58;76] | **1.15 [1.07; 1.24]** | 78 [67;86] | 69 [59;79] | **1.16 [1.07; 1.24]** |
| Female sex | 463 (37.2%) | 191 (32.1%) | 1.08 [0.84; 1.38] | 317 (41.8%) | 337 (31.2%) | **0.78 [0.62; 0.98]** |
| Living in French/Italian speaking cantons (vs. Swiss German) | 323 (25.9%) | 143 (24%) | 0.94 [0.73; 1.20] | 197 (26%) | 269 (24.9%) | 0.97 [0.76; 1.23] |
| Living in urban region | 948 (76.1%) | 443 (74.5%) | 1.06 [0.83; 1.35] | 583 (76.8%) | 808 (74.7%) | 1.02 [0.80; 1.30] |
| Annual deductible > 500 Swiss Francs | 146 (11.7%) | 94 (15.8%) | 0.82 [0.60; 1.12] | 73 (9.6%) | 167 (15.4%) | 0.93 [0.67; 1.31] |
| Having a managed care contract | 465 (37.3%) | 276 (46.4%) | 1.20 [0.97; 1.48] | 269 (35.4%) | 472 (43.7%) | 1.07 [0.87; 1.32] |
| Inpatient stays prior to index date | 348 (28%) | 80 (13.4%) | 0.75 [0.55; 1.02] | 255 (33.6%) | 173 (16%) | **0.73 [0.56; 0.96]** |
| High outpatient medication costs prior to index date | 121 (9.7%) | 33 (5.5%) | 1.11 [0.68; 1.81] | 87 (11.5%) | 67 (6.2%) | 1.02 [0.66; 1.56] |
| Prior exposure to secondary prophylactic drugs | 939 (75.4%) | 359 (60.3%) | 1.00 [0.55; 1.81] | 614 (80.9%) | 684 (63.3%) | 0.81 [0.46; 1.45] |
|  |  |  |  |  |  |  |
| Comorbidities (based on pharmaceutical cost groups) |  |  |  |  |  |  |
| Median number of chronic comorbidities | 3 [2;3] | 3 [2;3] | **1.24 [1.01; 1.52]** | 3 [2;3] | 3 [2;3] | **1.69 [1.38; 2.07]** |
| Acid related disorders | 365 (29.3%) | 97 (16.3%) | **0.68 [0.51; 0.92]** | 257 (33.9%) | 205 (19%) | **0.68 [0.52; 0.89]** |
| Bone diseases (osteoporosis) | 64 (5.1%) | 20 (3.4%) | 1.08 [0.60; 1.93] | 43 (5.7%) | 41 (3.8%) | 1.15 [0.69; 1.92] |
| Cancer | 27 (2.2%) | 6 (1%) | 0.64 [0.25; 1.66] | 14 (1.8%) | 19 (1.8%) | 1.42 [0.66; 3.06] |
| Cardiovascular diseases | 982 (78.9%) | 379 (63.7%) | 0.97 [0.52; 1.79] | 638 (84.1%) | 723 (66.9%) | 0.93 [0.50; 1.72] |
| Type 1 or type 2 diabetes | 289 (23.2%) | 93 (15.6%) | **0.74 [0.55; 0.99]** | 185 (24.4%) | 197 (18.2%) | 0.84 [0.64; 1.10] |
| Hypertension | 541 (43.5%) | 168 (28.2%) | **0.61 [0.47; 0.79]** | 363 (47.8%) | 346 (32%) | **0.59 [0.46; 0.75]** |
| Dementia | 63 (5.1%) | 17 (2.9%) | 0.83 [0.46; 1.50] | 44 (5.8%) | 36 (3.3%) | 0.85 [0.51; 1.40] |
| Epilepsy | 106 (8.5%) | 25 (4.2%) | 0.65 [0.40; 1.07] | 69 (9.1%) | 62 (5.7%) | 0.90 [0.59; 1.35] |
| Glaucoma | 119 (9.6%) | 48 (8.1%) | 1.28 [0.87; 1.89] | 84 (11.1%) | 83 (7.7%) | 0.90 [0.63; 1.29] |
| Gout/Hyperuricemia | 98 (7.9%) | 24 (4%) | 0.70 [0.43; 1.15] | 69 (9.1%) | 53 (4.9%) | 0.68 [0.45; 1.04] |
| Human Immunodeficiency Virus | 9 (0.7%) | 2 (0.3%) | 0.26 [0.05; 1.37] | 9 (1.2%) | 2 (0.2%) | **0.06 [0.01; 0.31]** |
| Intestinal inflammatory diseases | 11 (0.9%) | 1 (0.2%) | 0.22 [0.03; 1.83] | 9 (1.2%) | 3 (0.3%) | 0.26 [0.07; 1.05] |
| Iron deficiency anemia | 76 (6.1%) | 15 (2.5%) | 0.75 [0.41; 1.39] | 60 (7.9%) | 31 (2.9%) | 0.69 [0.42; 1.14] |
| Pain | 349 (28%) | 104 (17.5%) | 0.89 [0.65; 1.21] | 238 (31.4%) | 215 (19.9%) | 0.89 [0.68; 1.18] |
| Parkinson | 46 (3.7%) | 8 (1.3%) | 0.54 [0.24; 1.20] | 32 (4.2%) | 22 (2%) | 0.70 [0.39; 1.29] |
| Psychological disorders (sleep disorder, depression) | 314 (25.2%) | 118 (19.8%) | 1.18 [0.88; 1.57] | 213 (28.1%) | 219 (20.3%) | 1.06 [0.81; 1.39] |
| Respiratory illness (asthma, COPD) | 319 (25.6%) | 131 (22%) | 1.13 [0.86; 1.49] | 195 (25.7%) | 255 (23.6%) | 1.22 [0.94; 1.58] |
| Rheumatologic conditions | 73 (5.9%) | 18 (3%) | 0.79 [0.44; 1.43] | 59 (7.8%) | 32 (3%) | **0.54 [0.33; 0.91]** |
| Psychoses | 230 (18.5%) | 87 (14.6%) | 0.92 [0.68; 1.24] | 156 (20.6%) | 161 (14.9%) | 0.79 [0.60; 1.04] |
| Thyroid disorders | 70 (5.6%) | 26 (4.4%) | 1.26 [0.76; 2.10] | 50 (6.6%) | 46 (4.3%) | 1.02 [0.65; 1.62] |
|  |  |  |  |  |  |  |
| Anticoagulation treatments received prior to index date |  |  |  |  |  |  |
| Athrombin | 165 (13.3%) | 45 (7.6%) | 0.92 [0.62; 1.37] | 118 (15.5%) | 92 (8.5%) | 0.82 [0.58; 1.15] |
| Heparin | 105 (8.4%) | 21 (3.5%) | 0.66 [0.39; 1.12] | 72 (9.5%) | 54 (5%) | 0.97 [0.63; 1.49] |
| Vitamin K antagonists | 144 (11.6%) | 30 (5%) | 0.70 [0.45; 1.10] | 117 (15.4%) | 57 (5.3%) | **0.46 [0.32; 0.68]** |
| Other anticoagulation drugs | 7 (0.6%) | 2 (0.3%) | 0.94 [0.18; 4.93] | 5 (0.7%) | 4 (0.4%) | 1.15 [0.28; 4.74] |

***Supplementary table 3: comparison of medication expenditures between compliers and non-compliers***

*Amounts represent Swiss francs (CHF)*

|  | **4-class combination (main analysis)** | **3- or 4-class combination (sensitivity analysis)** |
| --- | --- | --- |
| **Full population (n=1840)** |  |  |
| Compliers Median [IQR] | 2,622 [2,281; 2,964]  (n=595) | 2,521 [2,291; 2,752]  (n=1081) |
| Non-compliers Median [IQR] | 2,597 [2,358; 2,837]  (n=1245) | 2,725 [2,380; 3,070]  (n=759) |
| TwoPM, unweighted Predicted difference [95%CI] | 509 [189; 829] | 336 [20; 652] |
| TwoPM, IPTW  Predicted difference [95%CI] | -12 [-497; 474] | -103 [-551; 345] |
| **Not pre-exposed (n=542)** |  |  |
| Compliers Median [IQR] | 2,287 [1,600; 2,973]  (n=236) | 1,987 [1,561; 2,413]  (n=397) |
| Non-compliers Median [IQR] | 1,606 [1,322; 1,889]  (n=306) | 1,670 [1,168; 2,171]  (n=145) |
| TwoPM, unweighted Predicted difference [95%CI] | 636 [263; 1,010] | 458 [56; 860] |
| TwoPM, IPTW  Predicted difference [95%CI] | 485 [-512; 1,482] | 481 [-296; 1,257] |

Abbreviations: TwoPM: Two-part model; IPTW: Inverse probability of treatment weights; IQR: Interquartile Range; 95% CI: 95% Confidence Intervals
