# Supplementary figures and images for: Going beyond the mean: economic benefits of myocardial infarction secondary prevention
Source: BMC Health Serv Res. 2020 Dec 4;20:1125. doi: 10.1186/s12913-020-05985-x (PMC7718707; doi:10.1186/s12913-020-05985-x)

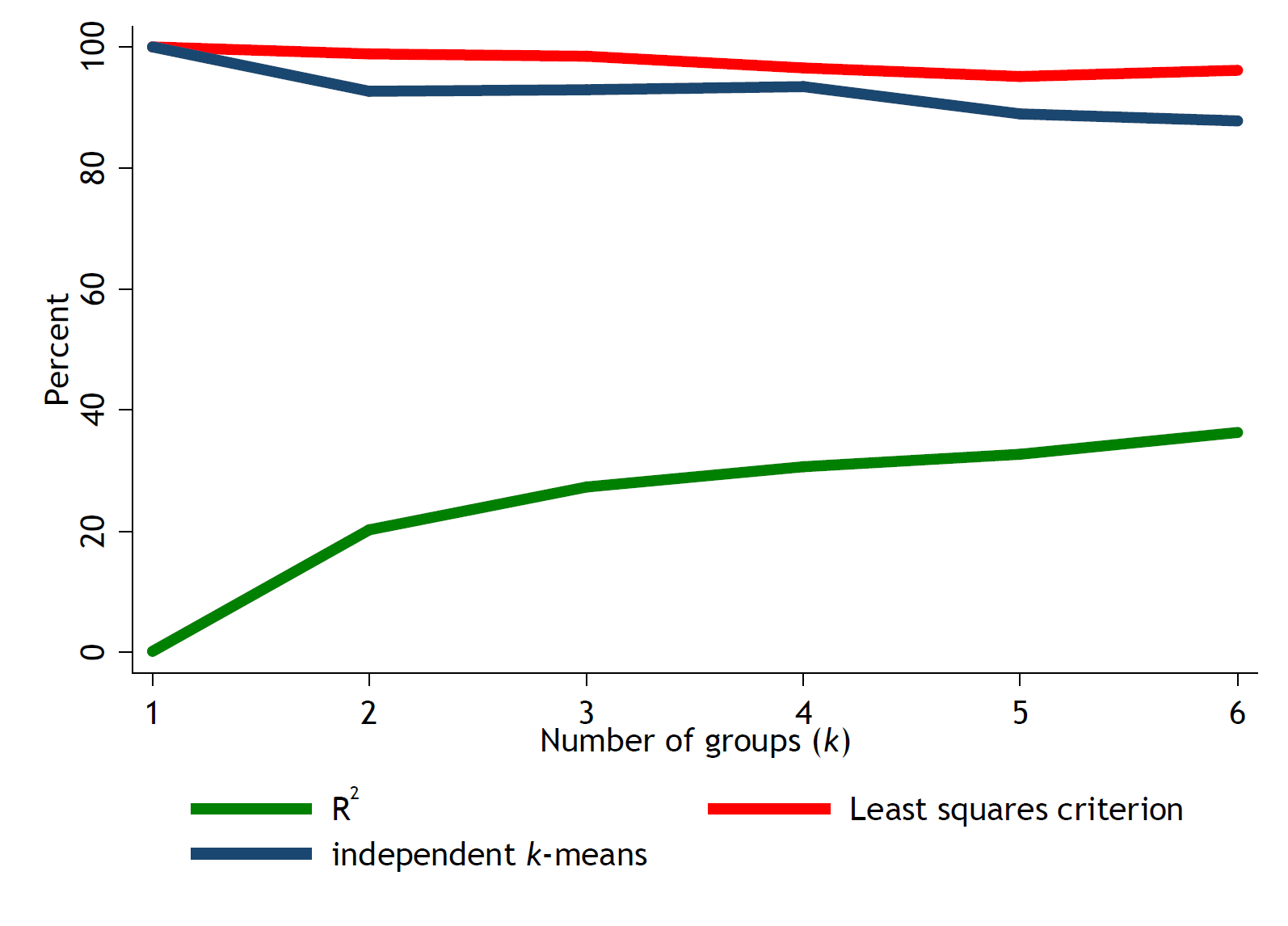

Supplement: Supplementary file 1 — Additional file 1: Supplementary figure legend 1. Indicators for trajectory model choice with respect to hypothesized number of distinct trajectory groups. The blue line indicates, for the validation data, the overlap between the training-based predictor and an independent k-means classification. Overall, classification overlap was in the order of 90% to 95% and peaked when four k-means groups were chosen. Supplementary figure legend 2. Cost decomposition by groups derived from the trajectory analysis, full sample. Supplementary figure legend 3. Cost decomposition by groups derived from the trajectory analysis, unexposed persons only. Supplementary Table 1. Comparison of clinical outcomes between compliers and non-compliers during observation period. Supplementary Table 2. Factors associated with compliance to 4-class secondary myocardial infarction prophylaxis (main analysis) or 3- or 4-class prophylaxis (sensitivity analysis). The results from the multivariable logistic regression model were used to calculate the inverse probability weights. Confidence intervals printed in bold face do not include 1, which indicates statistical significance at the 5% level. Supplementary Table 3. comparison of medication expenditures between compliers and non-compliers. [file 12913_2020_5985_MOESM1_ESM.zip › supplementary_figureR2_1.tif]

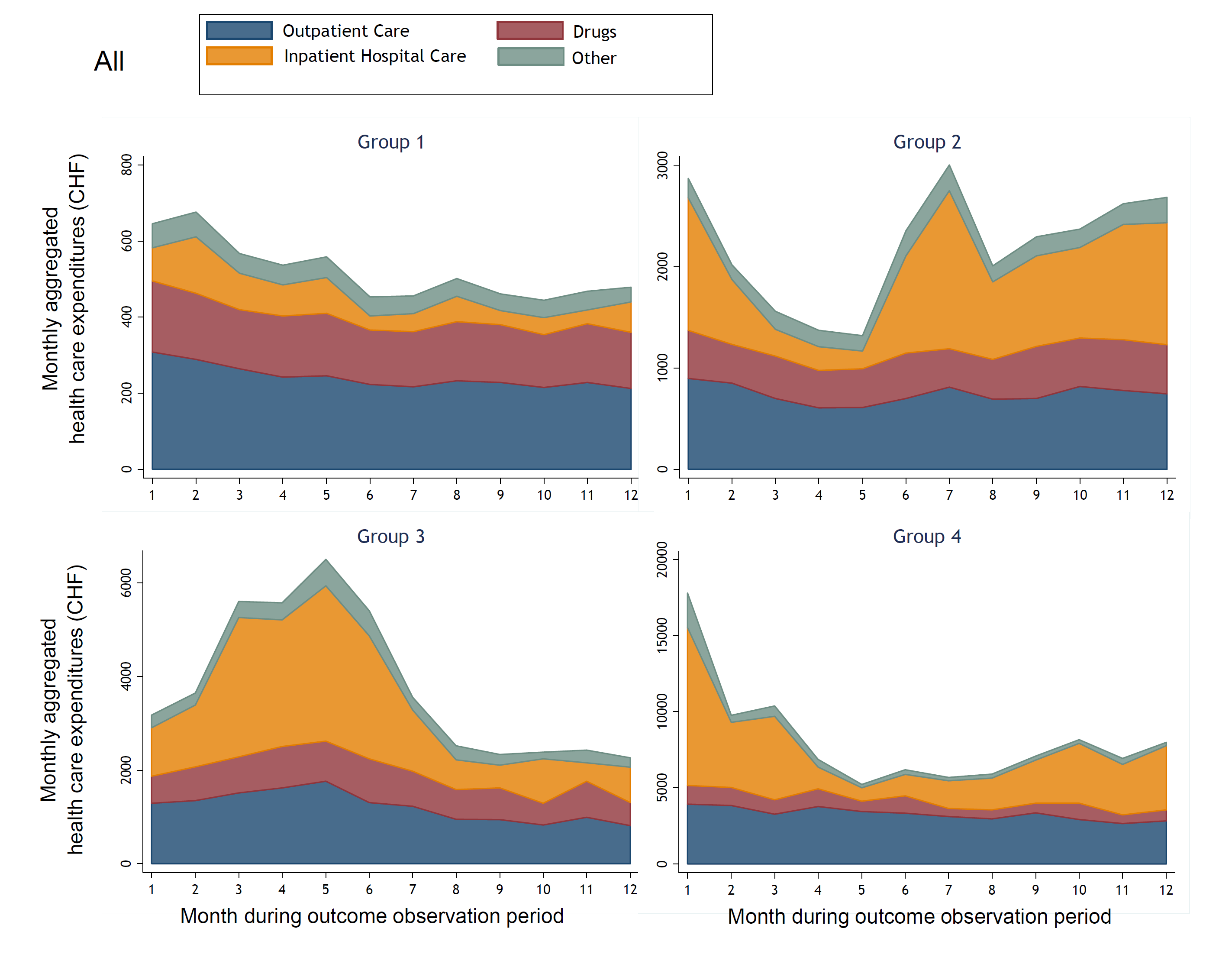

Supplement: Supplementary file 1 — Additional file 1: Supplementary figure legend 1. Indicators for trajectory model choice with respect to hypothesized number of distinct trajectory groups. The blue line indicates, for the validation data, the overlap between the training-based predictor and an independent k-means classification. Overall, classification overlap was in the order of 90% to 95% and peaked when four k-means groups were chosen. Supplementary figure legend 2. Cost decomposition by groups derived from the trajectory analysis, full sample. Supplementary figure legend 3. Cost decomposition by groups derived from the trajectory analysis, unexposed persons only. Supplementary Table 1. Comparison of clinical outcomes between compliers and non-compliers during observation period. Supplementary Table 2. Factors associated with compliance to 4-class secondary myocardial infarction prophylaxis (main analysis) or 3- or 4-class prophylaxis (sensitivity analysis). The results from the multivariable logistic regression model were used to calculate the inverse probability weights. Confidence intervals printed in bold face do not include 1, which indicates statistical significance at the 5% level. Supplementary Table 3. comparison of medication expenditures between compliers and non-compliers. [file 12913_2020_5985_MOESM1_ESM.zip › supplementary_figureR2_2.tif]

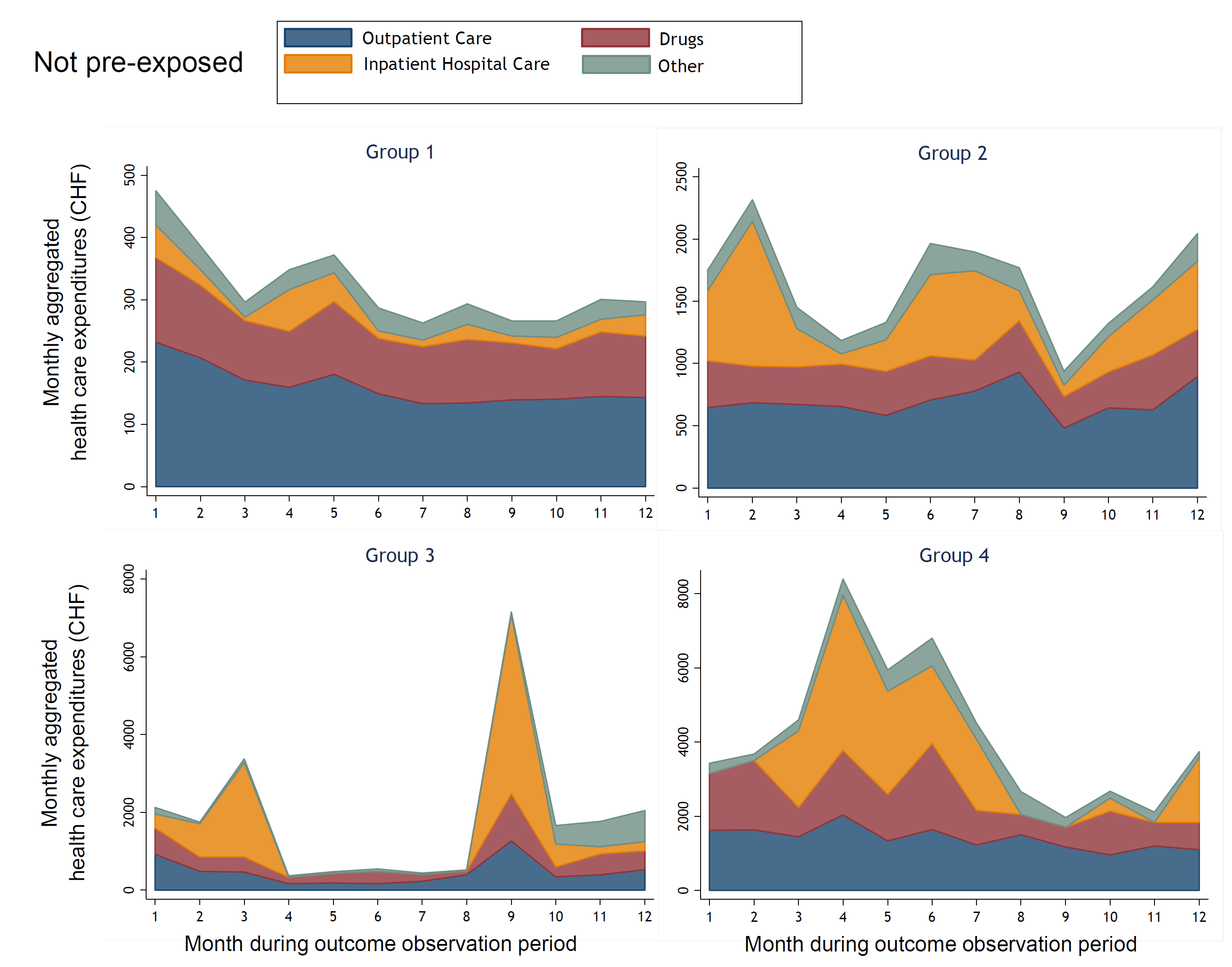

Supplement: Supplementary file 1 — Additional file 1: Supplementary figure legend 1. Indicators for trajectory model choice with respect to hypothesized number of distinct trajectory groups. The blue line indicates, for the validation data, the overlap between the training-based predictor and an independent k-means classification. Overall, classification overlap was in the order of 90% to 95% and peaked when four k-means groups were chosen. Supplementary figure legend 2. Cost decomposition by groups derived from the trajectory analysis, full sample. Supplementary figure legend 3. Cost decomposition by groups derived from the trajectory analysis, unexposed persons only. Supplementary Table 1. Comparison of clinical outcomes between compliers and non-compliers during observation period. Supplementary Table 2. Factors associated with compliance to 4-class secondary myocardial infarction prophylaxis (main analysis) or 3- or 4-class prophylaxis (sensitivity analysis). The results from the multivariable logistic regression model were used to calculate the inverse probability weights. Confidence intervals printed in bold face do not include 1, which indicates statistical significance at the 5% level. Supplementary Table 3. comparison of medication expenditures between compliers and non-compliers. [file 12913_2020_5985_MOESM1_ESM.zip › supplementary_figureR2_3.tif]
